# The NIH Toolbox for cognitive surveillance in Duchenne muscular dystrophy

**DOI:** 10.1002/acn3.50867

**Published:** 2019-08-31

**Authors:** Mathula Thangarajh, Aaron J. Kaat, Genila Bibat, Jennifer Mansour, Katherine Summerton, Anthony Gioia, Carly Berger, Kristina K. Hardy, Kathryn R. Wagner

**Affiliations:** ^1^ Department of Neurology Children’s National Health System District of Columbia Washington; ^2^ Department of Medical Social Sciences Northwestern University Chicago Illinois; ^3^ Center for Genetic Muscle Disorders Kennedy Krieger Institute, Johns Hopkins School of Medicine Baltimore Maryland; ^4^ Departments of Psychiatry & Behavioral Science and Pediatrics George Washington University School of Medicine District of Columbia Washington

## Abstract

**Objective:**

We performed a prospective, cross‐sectional cognitive assessment in subjects with Duchenne Muscular Dystrophy (DMD) and their biological mothers.

**Methods:**

Thirty subjects with out‐of‐frame mutations in the dystrophin (*DMD)* gene, and 25 biological mothers were evaluated using the National Institutes of Health Toolbox Cognition Battery (NIHTB‐CB). A parent completed the Behavior Rating Inventory of Executive Functioning (BRIEF), a standardized rating scale of executive functioning, for their child. Mothers completed self‐reports of BRIEF and Neuro Quality‐of‐Life (NeuroQoL) Cognitive Function.

**Results:**

Overall, the subjects with *DMD* scored approximately one standard deviation (SD) below age‐corrected norms on the NIHTB‐CB Total Cognition score. They scored 1.5 SD below age‐corrected norms in Fluid Cognition, which evaluates the cognitive domains of executive function, working memory, episodic memory, attention, and processing speed. Their performance was consistent with age expectations (i.e., within 1 SD below age‐corrected norms) in Crystalized Cognition, which evaluates vocabulary and reading. Subjects with *DMD* had higher T‐scores in several domains of BRIEF, demonstrating greater difficulty in executive functioning. The biological mothers had overall average or above average T‐scores on NIHTB‐CB. Mothers who were carriers of *DMD* mutation performed lower overall compared to mothers who were not carriers of *DMD* mutation (Cohen’s *d* = −1.1). Carrier mothers performed lower than average (1.5 SD) in Executive Function, measured by Flanker Inhibitory Control and Attention. Biological mothers scored within expected score ranges for adults in BRIEF and NeuroQoL.

**Interpretation:**

The NIHTB‐CB, combined with standardized self‐reported measures, can be a sensitive screening tool for cognitive surveillance in DMD.

## Introduction

Cognitive health is increasingly recognized as an area of unmet clinical and research need in Duchenne Muscular Dystrophy (DMD), a severely disabling neurological disease affecting the brain, skeletal, and cardiac muscles.^1^ DMD is due to the complete absence of dystrophin, encoded by the *DMD* gene, located on X‐chromosome.^2^ The spectrum of cognitive issues in DMD include a downward shift in intelligence, deficits in executive function and working memory, speech delay, learning difficulties, and social anxiety problems.^3^, ^4^, ^5^, ^6^, ^7^, ^8^, ^9^ There is also an increased incidence of neurodevelopmental disorders such as obsessive‐compulsive disorder (OCD),^10^ attention‐deficit hyperactivity disorder (ADHD),^10^, ^11^ and autism spectrum disorder (ASD)^11^, ^12^ ADHD is twice more common and ASD is four times more common in DMD than in the general population.^10^, ^11^, ^12^ Likewise, speech delay is 10 times more common in DMD.^8^

Cognitive problems in DMD begin early and persist. A 1‐year prospective, observational study of young boys with DMD showed lower composite score of cognition at baseline and on follow‐up, compared to typically developing peers.^13^ Cheiffo et al. evaluated approximately 41 boys with DMD longitudinally at preschool and school ages, and found that the boys who had hearing and speech issues at preschool had lower verbal intelligence at school age, compared to those boys who did not have hearing and speech issues.^14^ The cumulative comorbidities in DMD is high. ADHD and OCD commonly co‐occur in DMD.^10^, ^15^ The effect of these cognitive problems are a significant source of morbidity with long‐term, educational, vocational, and social implications.

The cognitive health of women carriers with *DMD* mutations have not yet been evaluated. Subtle executive dysfunction have been reported in other X‐linked disorders, namely, in premutation carriers of Fragile X tremor/ataxia syndrome^16^ and ornithine transcarbamylase deficiency (OTCD), an urea cycle disorder.^17^ The evaluation of cognitive health in women carriers of *DMD* mutation not only informs neurobiological mechanisms that underpin higher order cognitive skills, but also has practical implications for anticipatory guidance.

The Center for Disease Control (CDC)‐supported DMD Care Considerations encourage mental health and quality‐of‐life screening at each neuromuscular clinic visit.^18^ Additionally, neuropsychological evaluations are recommended within the first year of diagnosis, when cognitive and behavioral concerns are first identified, and periodically as needed to track functioning.^18^ Yet, data from epidemiological studies^19^, ^20^ and healthcare provider‐surveys^21^ demonstrate significant barriers that families face with adherence to standard‐of‐care guidelines. Access to specialty services and out‐of‐pocket expenses for families are key barriers to adherence.^19^ Of note, only 24% of muscular dystrophy subjects had had a neuropsychological assessment in accordance with DMD Care Considerations.^19^ Furthermore, caregivers identified unmanaged psychosocial issues as an area of unmet need.^20^ A pertinent finding from the Neuromuscular Disease Healthcare Provider Survey found that psychological health was the “number one” area of high unmet need in neuromuscular disorders, affecting nearly 90% of patients, and more than 50% of the healthcare providers reported that access to a mental health professional as extremely challenging.^21^

To meet this critical need for cognitive and mental health surveillance in neurological diseases, new modes of assessment are necessary. The use of a technology‐enhanced, reliable cognitive assessment tool that is readily available to medical team members may mitigate access barriers that families with neuromuscular disorders face, while closing the gap between standard‐of‐care guidelines and clinical adherence. Toward this goal, we assessed the feasibility of the National Institutes of Health (NIH) Toolbox for the Assessment of Neurological and Behavioral Function Cognition Battery (NIHTB‐CB) as a screening tool for cognitive assessment in DMD. The NIH Toolbox is a scientifically rigorous, multidimensional measure designed to bring uniformity to evaluation of neurological function across the life span.^22^, ^23^ The NIHTB‐CB tests have previously been evaluated for their reliability, validity, and other psychometric properties,^24^, ^25^, ^26^ including among individuals with several different neurodevelopmental disorders.^27^ We hypothesized that the NIHTB‐CB would be sensitive to detect cognitive deficits in domains known to be impaired in DMD. In addition, we hypothesized that *DMD* carrier women may experience subclinical cognitive problems, compared to *DMD* noncarrier women. We were interested in combining performance‐based testing with standardized person‐centered self‐ or proxy‐report measures to help identify those who may experience greater impact on adaptive functioning and quality‐of‐life.

## Subjects and Methods

### Study design

This prospective study enrolled subjects from two regional academic institutions. Institutional Review Boards (CNMC#9965 and JHU/KKI#00135833) approved the study at both institutions. All study participants provided written informed consent; additionally, subjects between ages 13 and 17 years provided written assent. The study was conducted in accordance with the Declaration of Helsinki (2000) and was fully compliant with the Principles of Good Clinical Practice according to the International Conference on Harmonization.

### Study subjects

Study subjects were children with a confirmatory genetic testing that showed out‐of‐frame mutation in *DMD* gene, and their biological mothers. We evaluated a total of 55 subjects, including 29 boys with DMD, 1 girl who was evaluated for learning and behavioral difficulties and was found to have out‐of‐frame mutation in *DMD*, and 25 biological mothers. Of the total 55 subjects, 25 were mother‐son dyads. There were two sets of twin boys. Three biological mothers were not evaluated; one subject was adopted, one subject was conceived through third‐party reproduction, and one biological mother had schedule conflict. The children received a nominal gift card for study participation.

### Study measures

#### NIHTB‐CB

Subjects completed age‐appropriate instruments of the NIHTB‐CB in approximately 35 min, on an iPad. The NIHTB‐CB^24^, ^25^ consists of seven tasks that assess core cognitive domains, and which provide composite scores for total, fluid, and crystallized cognitive abilities. Fluid Cognition is obtained through evaluation of executive function (Dimensional Change Card Sort (DCCS)), episodic memory (Picture Sequence Memory (PSM)), working memory (List Sorting Working Memory (LSWM)), processing speed (Pattern Comparison (PCPS)), and attention (Flanker Inhibitory Control and Attention). Crystalized Cognition includes assessment of reading and vocabulary. The Total Cognition score is a normalized average of Fluid and Crystalized Cognition scores. Age‐corrected standard score norms for children (mean 100, SD 15), and demographically adjusted T‐score norms for biological mothers (age, sex, race/ethnicity, and years of education) (mean 50, SD 10) were obtained based on individual performance that compared subject’s score with population‐normed score.^28^ Higher scores on the NIHTB‐CB are indicative of better functioning. The NIHTB‐CB was administered by personnel who had completed appropriate level of training. All subjects completed NIHTB‐CB in English language.

#### Behavior rating inventory of executive functioning (BRIEF)

A parent completed BRIEF questionnaire, a widely used assessment of executive functioning for children ages 5–18 years.^29^ BRIEF has 86 items that assess Behavioral Regulation, Metacognition, and General Executive Composite (GEC). Parents are asked to consider the frequency with which each item has been a problem over the last 6 months, responding on a 3‐point Likert scale consisting of “never,” “sometimes,” and “often.” The items on BRIEF are ecologically valid behavioral correlates to presumed neurocognitive difficulties with executive functioning. The psychometric properties of BRIEF are strong using normative samples (1419 parents) weighted to match ethnic and gender proportions in the U.S. population with high internal consistency (Cronbach’s alpha range = 0.73–0.90) and strong validity and test–retest reliability (0.79–0.88). The BRIEF thus provides parent and self‐reported outcomes of problems related to attention, memory, and executive function that occur in everyday life. Raw scores are linear transformed into T scores (mean = 50, SD = 10); higher scores indicate greater difficulties. All participants completed BRIEF in English language. Of the 30 children evaluated, 28 had BRIEF data, and data were not available in the other two subjects. There was no missing data on test items from any of the subjects. Biological mothers completed a self‐report Adult Version of BRIEF (BRIEF‐A).^30^

#### NeuroQoL measure

Biological mothers completed a 14‐item custom short form from the Cognitive Function, version 2.0 item bank. NeuroQoL measures are rated using a 5‐point scale. The raw score is converted into a standardized T‐score (mean of 50, SD 10).^31^ Higher scores are indicative of better cognitive function. Data in NeuroQoL was available in 21 of the 25 biological mothers. There was no missing data on any of the items for the mothers.

### Demographic and medical information

A parent provided information regarding several aspects of their child’s medical care and education, including the type of oral corticosteroid prescribed by the medical team (prednisone versus deflazacort), any change in corticosteroid dose in the last 3 months prior to study assessment, other concurrent medication for ADHD, and any change in individualized educational plan for each child. Biological mothers completed a study questionnaire regarding their own medical and behavioral health, genetic testing for *DMD* carrier status, and any medication use.

### Statistical analyses

Descriptive profiles were generated for the NIHTB‐CB and the BRIEF. We calculated effect sizes between *DMD* carrier and *DMD* noncarrier mothers, as we rationalized that obtaining effect sizes will better inform future studies. Statistical analysis was performed using R software,^32^ and plots were generated utilizing the ggplot2 library.^33^

## Results

### Subject demographics

The mean age at study evaluation for the 30 children with DMD was 11.4 years (range 6–16 years). Of these, 18 had *DMD* deletions, 11 had *DMD* nonsense mutations, and 1 had *DMD* duplication. Seventeen children had mutations upstream of *DMD* exon 45, and 13 children had mutations downstream of *DMD* exon 45.

Data regarding co‐morbid conditions (ADHD, ASD, anxiety, depression) were available in 28 of the 30 subjects. Of the 28 subjects in whom data were available, five had a formal diagnosis of ADHD, and three were on medication for the same. None of the 28 subjects had a diagnosis of ASD. Anxiety was reported by two subjects, whereas most did not (*n* = 26).

Of the 30 subjects, 26 were currently on oral corticosteroids (19 on deflazacort, 6 prednisone, 1 in a double‐blind trial of deflazacort and prednisone). Two subjects were not on oral corticosteroids and data were missing in two subjects.

The mean age of the biological mothers was 41.7 years (range 29–52 years). Of the 25 mothers evaluated, 11 were *DMD* carriers, eight were noncarriers of *DMD*. Three mothers reported no knowledge regarding their *DMD* carrier status, and data were not available in three mothers. Demographic details are summarized in the Table 1.

**Table 1:** Subject characteristics included in the study.

Subject characteristics			
Children (*n* = 30)		Biological mothers (*n* = 25)	
Age, mean (range in years)	11.4 (6–16)	Age, mean (range in years)	41.7 (29–52)
Gender		DMD carriers	11
Male	29	DMD noncarriers	8
Female	1	Employment	
*DMD* mutation		Full‐time	14
Deletion	18		
Nonsense	11		
Duplication	1		
Race		Currently not employed	7
White	25	House‐hold income	
Asian	2	<50 K	5
Biracial	3	50–100 K	5
Ethnicity		100–150K	4
Not Hispanic	27	>150 K	8
Hispanic	3	Race	
Grade repetition		White	19
No	21	African‐American	1
Yes	5	Asian	1
Missing	4	Biracial	2
Co‐morbid conditions (*n* = 28)			
Attention‐deficit hyperactivity	5		
Anxiety	2		
Autism spectrum disorder	0		
Oral corticosteroid use (*n* = 28)			
Deflazacort	19		
Prednisone	6		
Double‐blind trial*	1		
Steroid‐naive	2		

* Participant is currently enrolled in a double‐blind trial of deflazacort versus prednisone.

### Cognitive profiles of the children with *DMD*

**Figure 1:**
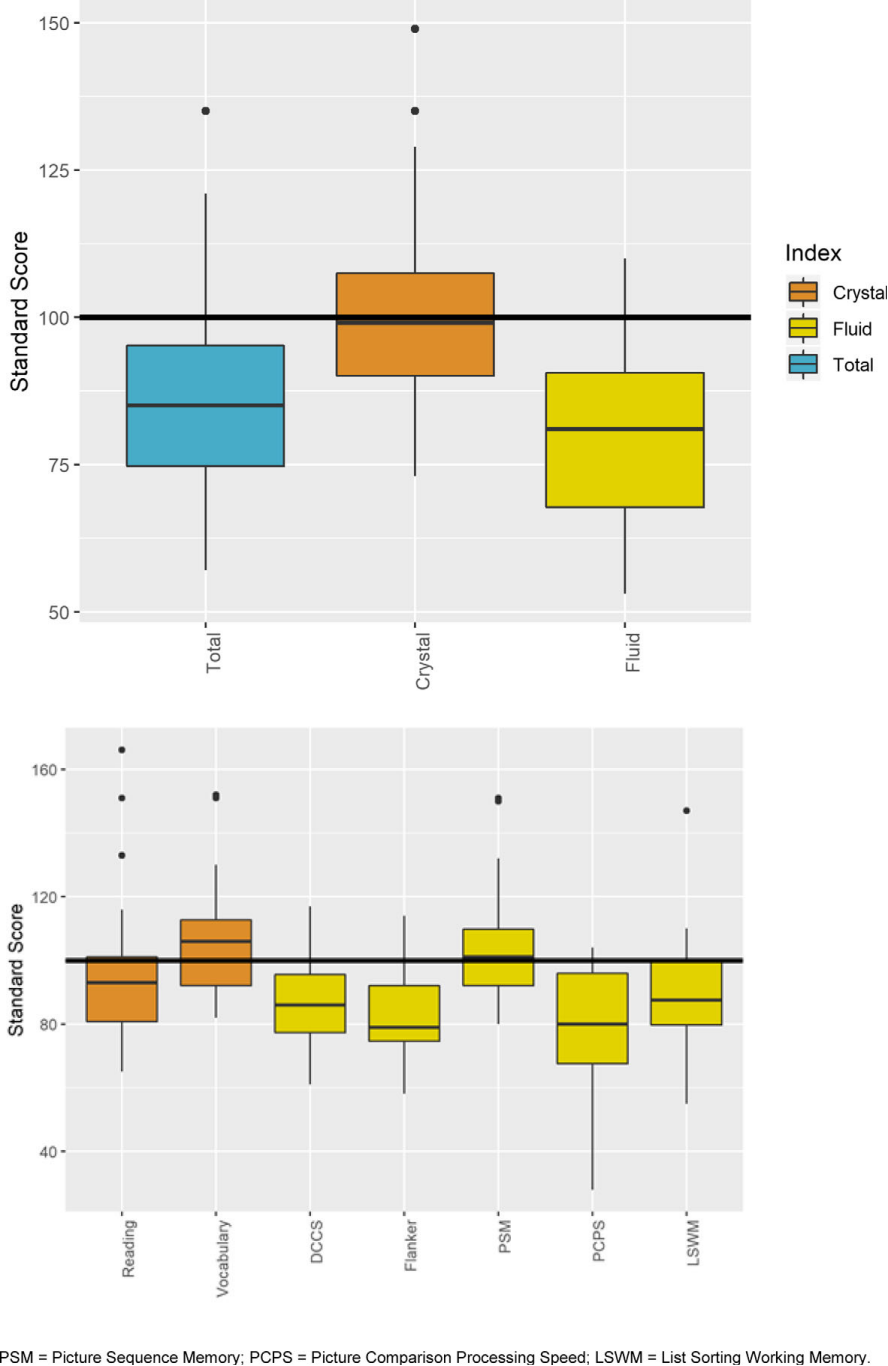
Performance of children with *DMD* mutations on cognitive domains as assessed by the NIH Toolbox‐Cognition Battery.

All 55 subjects completed the NIHTB‐CB without any interruption, showing good task‐specific effort, and easy comprehension of tasks. The overall NIHTB‐CB Total Cognition score was approximately 1 SD below age‐corrected norm in the 30 children evaluated (mean = 87, SD = 19). There was a distinct divergence of performance in fluid versus crystallized cognitive domains (Fig. 1, upper panel). Specifically, the children scored 1.5 SD below age expectations on the Fluid Cognition score, in contrast with average performance on Crystalized Cognition (mean = 101, SD = 19). The bifurcation of fluid and crystallized cognitive abilities is further demonstrated on individual subtests. Specifically, the children performed lowest on Flanker Inhibitory Control and Attention, PCPS, and LSWM, domains attributed to fluid cognitive skills (Fig. 1, lower panel). By contrast, performance in vocabulary and reading was at par with age expectations.

### Cognitive profile of carriers and noncarriers of *DMD* mutation

**Figure 2:**
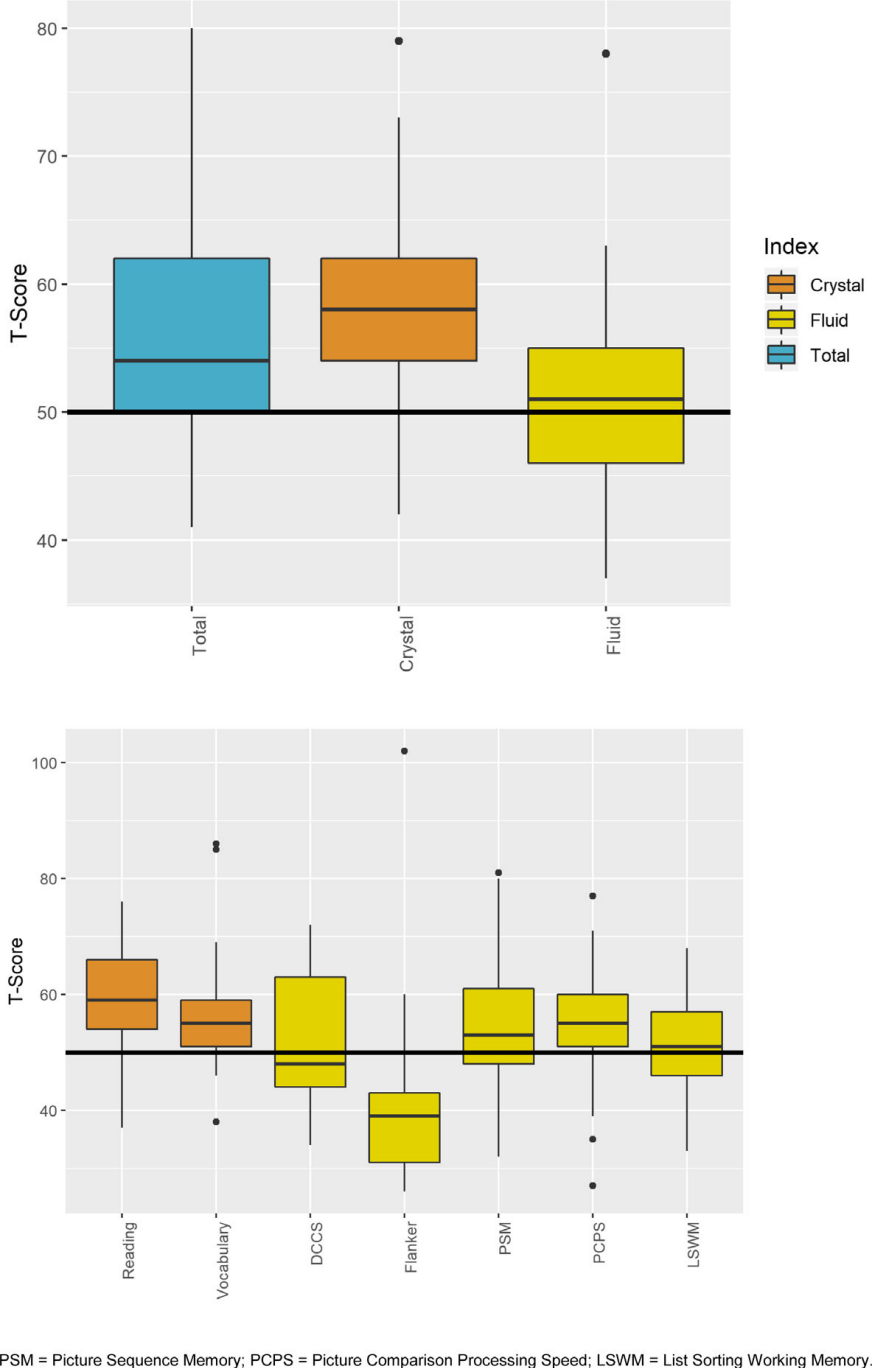
Performance of biological mothers of children with *DMD* mutations on cognitive domains as assessed by the NIH Toolbox‐Cognition Battery.

The 25 biological mothers had overall NIHTB‐CB Total Cognition T‐score in the average or above average range (mean = 56, SD = 9) (Fig. 2, upper panel). They performed overall better in crystallized cognitive domains compared to the fluid cognitive domains. On individual subtests within the fluid cognitive domain, they scored lowest in Flanker Inhibitory Control and Attention (mean = 41, SD = 16) followed by DCCS (mean = 52, SD = 12) (Fig. 2, lower panel). By contrast, performance in vocabulary (mean = 57, SD = 11) and reading (mean = 60, SD = 10) was above their population‐normed appropriate reference groups.

**Figure 3:**
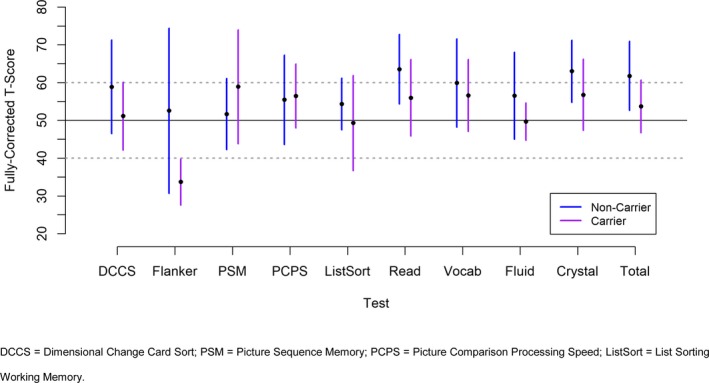
Cognitive profile between carriers and noncarriers of *DMD* mutation.

We compared the cognitive profile between carriers and noncarriers of *DMD* mutation (Fig. 3). Carriers of *DMD* mutation scored lower in Total, Crystalized, and Fluid composite scores (Cohen’s *d* = −1.0, −0.7, and −0.7, respectively), compared to noncarriers of *DMD* mutation. The most discrepant tests between carriers and noncarriers were Flanker Inhibitory Control and Attention, and Reading (Cohen’s *d* = 1.2 and −0.7 respectively). Carriers of *DMD* mutation scored lower than *DMD* noncarriers in most other tests of Fluid Cognition, but to a lesser degree. The exceptions were in PSM and PCPS; carriers of *DMD* scored higher than *DMD* noncarriers on PSM (Cohen’s *d* = 0.5) and in PCPS (Cohen’s *d* = 0.1).

### BRIEF in children with *DMD* and biological mothers

**Figure 4:**
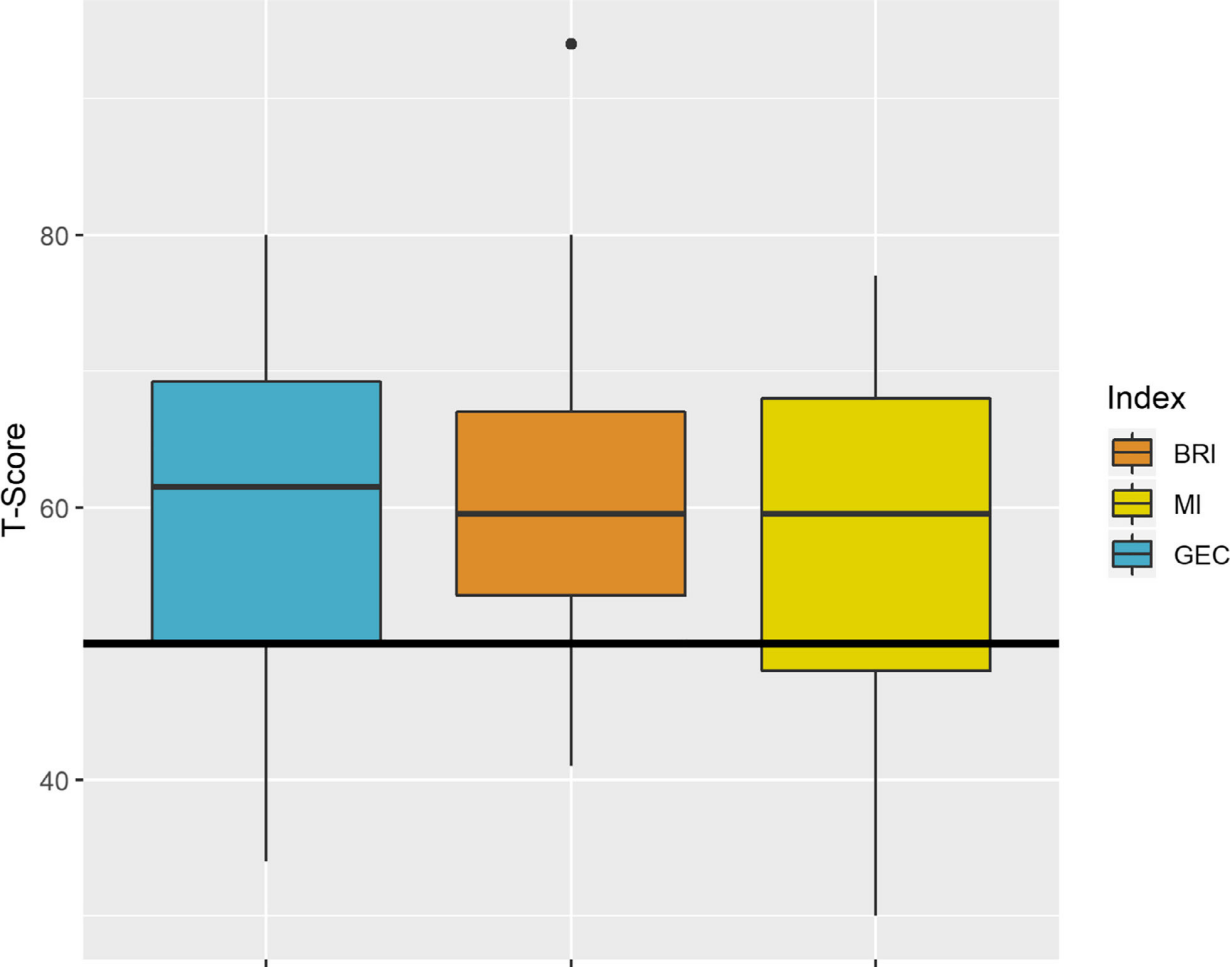
Behavior Rating Inventory of Executive Functioning (BRIEF) in children with *DMD* mutations.

The mean GEC score in children with *DMD* was 59.6 (SD 12.2, range 34–80) (*n* = 28). The children with DMD did not show a clear pattern of strength or weakness on GEC, Behavioral Regulation Index (BRI), and Metacognition Index (MI) (Fig. 4). On individual tests within the BRIEF MI, working memory was a clear weakness in *DMD* children (data not shown).

**Figure 5:**
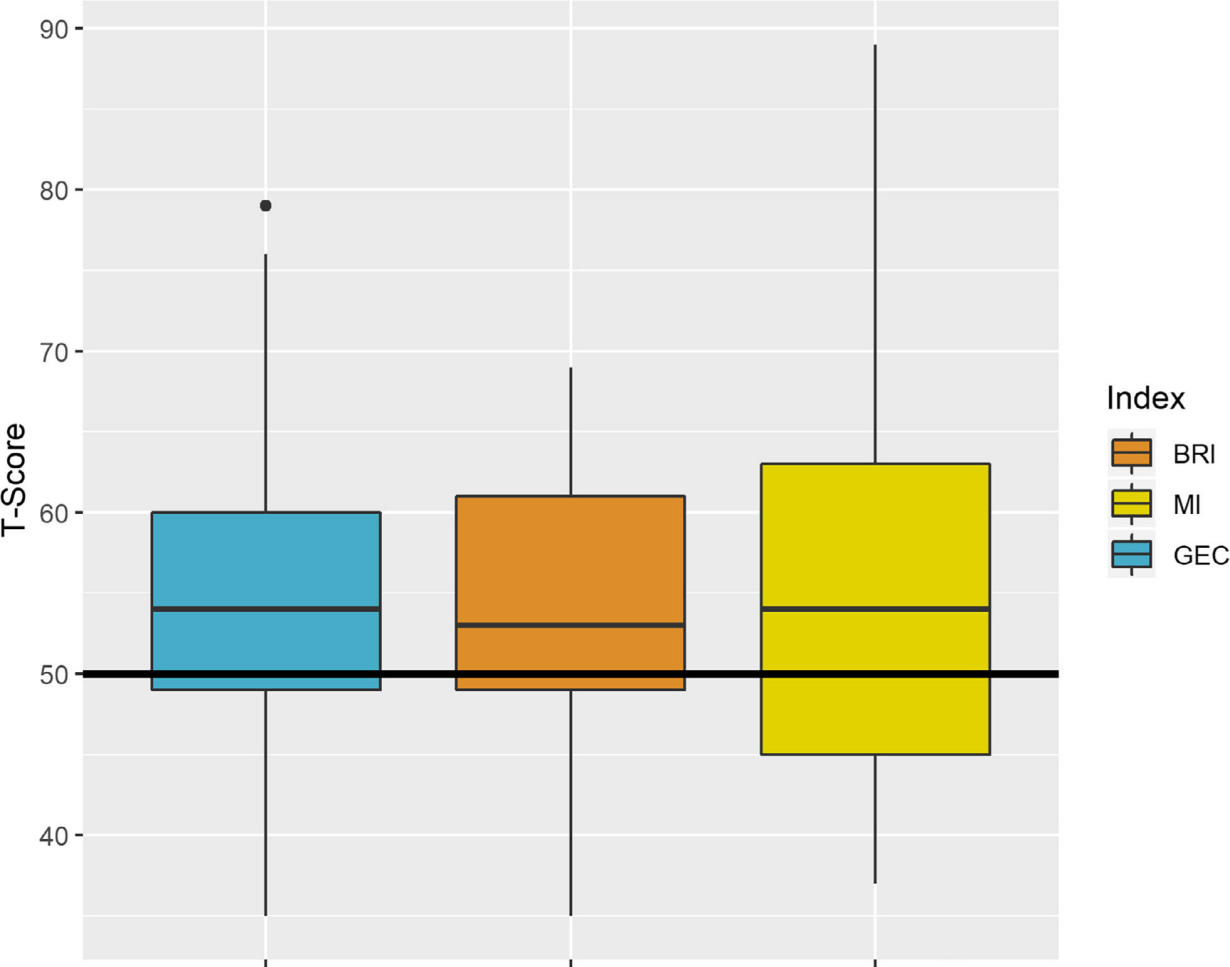
Behavior Rating Inventory of Executive Functioning (BRIEF) in biological mothers of children with *DMD* mutations.

Evaluation of BRIEF measures on biological mothers showed that nearly all scores were within expected limits (Fig. 5). In individual tests, working memory was a potential weakness, though the group averages remained below the cut‐off of clinical concern (i.e., a T‐score greater than 65) (data not shown).

### NeuroQoL measure of cognition

Twenty‐one mothers rated their own perceived cognitive functioning on the 14‐item custom short form. The mean score was 49 (SD 7.5) (*n* = 21) which is within the expected score range for adults.

## Discussion

Adherence to standard‐of‐care guidelines can be challenging for medical teams. In particular, resource constraints may lead medical teams to prioritize one service over the other, especially during lengthy multidisciplinary care visits. These system‐based challenges, including fewer mental health specialists being part of multidisciplinary medical teams, or long‐wait times for formal neuropsychological evaluation, are significant barriers to adherence to standard‐of‐care guidelines. This is especially true for cognitive health in DMD, where fewer than 25% of patients receive adequate evaluation of their cognitive health.^19^

Our primary goal with this pilot study was to evaluate the feasibility of using a technology‐enhanced developmental assessment tool for cognitive surveillance in DMD. We chose the NIHTB‐CB for the following rationale: (1) the availability of population‐normed data, (2) assessment across the life‐span (ages 3–85 years), (3) ability to control for education for adults or maternal education for children, (4) portability (available as an iPad‐app), and (5) brevity of assessment duration (approximately 30 min), a valuable feature should the multidisciplinary team elect to use in clinic. The technology‐enhanced platform permits the NIHTB‐CB to be user‐friendly and nonreliant on highly trained professionals. We envision that the portability of NIHTB‐CB allows for evaluation of “every patient, everywhere” improving the “global footprint” of cognitive surveillance in DMD.

Our data indicate that the NIHTB‐CB identifies cognitive domains specifically affected in DMD. Notably, while children with *DMD* mutations had overall average scores, the NIHTB‐CB was able to discriminate between relative strengths and weaknesses. For example, the NIHTB‐CB successfully captured the relative deficits in executive function, working memory, and attention (DCCS, LSWM, Flanker Inhibitory Control) among the subjects. These cognition functions are known to be affected in DMD, as documented from earlier studies, which has shown lower intellect with disproportionate deficits in executive function and working memory in DMD.^6^, ^34^, ^35^

We also observed that parents were likely to report that their child had relative difficulty with working memory on BRIEF. These data suggest that parents appreciate that there is an impact on day‐to‐day functioning, and that some children experience greater difficulty compared to others.

A novel finding of our study is the observed cognitive vulnerability in carriers of *DMD* mutation. In particular, women carriers of *DMD* performed lowest in the task of working memory and attention, compared to noncarrier mothers on population‐normed data. Sprouse et al. assessed asymptomatic and symptomatic OTCD subjects on tasks assessing executive functioning. When cognitively challenged, asymptomatic OTCD subjects performed poorly compared to symptomatic subjects on performance‐based tasks, but did not show statistically significant difference in BRIEF scores.^17^ An important distinction of our study is that none of the biological mothers self‐reported any cognitive concerns. Their scores on performance‐based tasks showed that they have normal cognitive reserve, but they performed sub‐par compared to noncarrier women drawn for this same study.

While the small sample size of the women who are *DMD* carriers in our study preclude definite conclusions, we hypothesize that skewed X‐inactivation in sexually dimorphic neural tissue may place carriers of *DMD* mutation more vulnerable to cognitive problems than noncarriers. This hypothesis is based on converging evidence of the role of the X‐chromosome in brain structure and function, and the genes that control executive function. An imaging study of 45, X females showed abnormally enlarged amygdala.^36^ A correlation between X‐chromosome deletion and brain imaging identified *Usp9x*, an X‐linked gene, which influences amygdala structure and function.^36^ Monoamine systems such as MAO‐B play important roles in attention and executive function, with depletion of monoamines leading to decreased attention on vigilance task.^37^ Females with 45, X demonstrate lower MAO‐B activity^38^, compared to females and males. The neural circuitry mechanisms that are affected by sexually dimorphic genes are yet to be fully characterized. Large studies in carriers of *DMD* mutation is necessary to understand fully these findings. Our preliminary finding that carriers of *DMD* mutation may exhibit cognitive vulnerability has a potential impact in anticipatory guidance of carriers of *DMD* mutation. Currently, anticipatory guidance is limited to periodic evaluation for cardiomyopathy. To our knowledge, this is the first study evaluating cognition in women with *DMD* mutation. Should our preliminary findings be replicated in a large sample, then anticipatory guidance for carriers of *DMD* mutation may potentially include cognitive evaluation.

We did not perform concurrently a full neuropsychological evaluation of our study subjects. This choice was deliberate. First, evaluation by a neuropsychologist is a potential access barrier, and may involve out‐of‐pocket expenses for the family. Second, neuropsychological evaluations are lengthy, expensive, and require highly trained professionals to administer, score, and interpret the results. Our goal with the NIHTB‐CB is that a qualified member of the multidisciplinary team can use it with ease for cognitive surveillance in DMD. We wish to emphasize that our envisioned use of the NIHTB‐CB in a clinical setting is not intended to substitute the services of a neuropsychologist or a professional mental health provider. Rather, we advocate for a tiered, risk‐adapted prevention‐based model of neuropsychological assessment and intervention in pediatric population with chronic medical conditions, including DMD.^39^ This model recommends universal monitoring followed by targeted screening and comprehensive evaluation. Our study supports that the NIHTB‐CB may be a tool that will allow for identification of “at‐risk” individuals, and prioritize timely evaluation and intervention.

Our study has some limitations, the first being the bias of convenient sampling. We approached families who were in clinic for routine neuromuscular visits with most of them electing to participate in the study. Second, our sample size (30 boys, 25 mothers) is relatively small. DMD is the most common X‐linked neuromuscular disorder but still a rare disorder. Third, we did not perform correlations between performance on NIHTB‐CB based on *DMD* mutation location, although we have earlier shown that those with nonsense mutations in 3’ of *DMD* show objective deficits in working memory.^35^ We were agnostic to *DMD* mutation location because of our intended goal of using the NIHTB‐CB as a cognitive surveillance tool in DMD. Last, we did not evaluate any social predictors of cognition. The NIH Toolbox is capable of adjusting for education (for adults) or maternal education for children (in both cases, education is a proxy for socio‐economic status), and other social predictors of cognition such as race/ethnicity, gender, and age.^28^ For the analyses of *DMD* carrier versus noncarrier mothers, we used these adjustments. However, for the analysis of the NIH Toolbox scores for boys, we did not consider adjustments beyond that of age (which is the standard practice for educational testing). Furthermore, we did not evaluate any other social predictors beyond the adjustments available within the NIH Toolbox, given the small sample size. While inclusion of social predictors is indeed important and relevant in this population, future studies are using large sample sizes that will help shape public health policy are needed. Despite the above mentioned limitations, we successfully demonstrate that the NIHTB‐CB is sensitive to identify cognitive deficits in DMD.

Based on age and manifestations of cognitive problems in early childhood, it is likely that the NIH Toolbox may be used for screening younger individuals. Currently, it is still unclear whether cognitive deficits are static or progressive in DMD. Boys with DMD perform poorly on measures of academic achievement compared to their unaffected siblings.^40^ These academic challenges are considered partly due to verbal working memory deficits that boys with DMD have. We would therefore recommend that medical teams caring for these individuals be vigilant about cognitive screening across the life‐span. The technology‐enhanced NIH Toolbox allows for flexibility in its use. Thus, in addition to initial screening and periodic surveillance, which can be individualized depending on the subject, the medical teams can also consider cognitive surveillance during developmental transition points such as moving to middle school or high school, when cognitive demands increase. Teams can collaborate with other medical providers and school and educational specialists to evaluate response to any intervention using the NIH Toolbox. Because the NIH Toolbox allows assessment across the life‐span (3+ to 80 years), it can allow confident longitudinal measurements of same domain constructs.

## Author Contributions

MT conceptualized and designed study; acquired and analyzed data and wrote the first draft of the manuscript. AJK was involved in data analysis, data visualization and revised the manuscript. GB data acquisition was involved in study oversight and data acquisition. KKH was involved in study design, data interpretation, and manuscript revision. KRW was involved in data acquisition, data interpretation, and manuscript revision. All authors approved the final version of the manuscript.

## Conflict of Interest

No relevant financial disclosures.
